# Nurses’ Awareness of Preterm Neonates’ Sleep in the NICU

**DOI:** 10.5539/gjhs.v8n6p226

**Published:** 2015-11-17

**Authors:** Nasrin Mahmoodi, Azizollah Arbabisarjou, Mahmood Rezaeipoor, Zahra Pishkar Mofrad

**Affiliations:** 1 Pregnancy Health Research Center, Zahedan University of Medical Sciences, Zahedan, Iran

**Keywords:** sleep, awareness, preterm neonates

## Abstract

**Introduction:**

Fetus and neonate spend most of their time sleeping inside and outside the womb. Sleep is considered a crucial action of neonatal period similar to breathing and nutrition. It plays a key role in brain development. Today, it is shown that sleep plays a predominant role in body temperature regulation, energy saving and neuronal detoxification. Sleep is the most important behavioral state of neonates, particularly in preterm ones. Noise, light, invasive treatment and caring activities are among disturbing factors in the neonatal intensive care unit (NICU) that leave negative impacts on brain development through disturbing the sleep process.

**Materials and Methods:**

This descriptive study assessed all NICU nurses of Ali-ibn-Abitaleb hospital using the census sampling method. Demographic data was collected through a questionnaire with 10 questions about active sleep (AS) cycles, also referred to as REM, methods for inducing AS and AS specifications in neonates. The questionnaire was distributed between the nurses. After completion, data was analyzed using SPSS 16 and descriptive statistics method.

**Findings:**

According to analyses, 24%, 20%, 48% and 92% of nurses gave correct answers to questions about AS cycle, AS in neonates, the role of sleep in saving energy and ideal noise level, respectively.

**Conclusion:**

According to results, nurses had a low level of knowledge towards neonatal sleep. All nurses need to know the importance of sleep in preterm neonates. The main role of inducing sleep is to protect the development of the neonates’ brain in the NICU. Those nurses who spend a remarkable portion of their time for caring neonates in the NICU play a significant role in neonatal sleep care.

## 1. Introduction

Sleep is one of the phenomena of the creation and a sign of divine wisdom. All entities need to rest in order to recover their energy and survive. Sleep is a great divine blessing resulting in physical and spiritual peace. Fetus and neonate spend most of their time sleeping inside and outside the womb. Sleep is considered a crucial action of neonatal period similar to breathing and nutrition. Improvement of the physical growth of neonates depends on sleep (Bertelle et al., 2007). Today, the rate of neonates referred to the NICU and demand intense cares due to prematurity or physical problems is increasing. In such a condition, meeting the necessary needs of such neonates is of a great importance. Sleep is an essential need of NICU neonates. They are in rapid brain growth stage and according to new studies, sleep plays a key role in brain development.

Neonates need to sleep in order to enable the growth of five sense and neural systems as well as the structural development of hippocampus, pons, brainstem and midbrain (Graven, 2006; Scher et al., 2005). Neonates have three sleep stages: quiet sleep (QS), also referred as NREM sleep, active sleep (AS), and indeterminate sleep (IS). The duration of QS increases as the control of neural system improves. This means that as preterm neonates grow over time, the duration of QS increases and turns into the most organized sleep state in neonates [1]. Neonates sleep between 16 hours to 18 hours daily with equispaced stages during days and nights. As age increases and circadian rhythm evolves, sleep is focused mostly in nights (Mirmiran et al., 2003).

During the neonatal period, AS plays a significant role in brain development and formation of synapse in both term and preterm neonates (Foreman, 2008). Sleep plays a vital role in the development of eyesight system in neonates (Dang-Vu et al., 2006). During sleep, the secretion of different hormones including melatonin and growth hormone increases. Moreover, the production of proteins is increased while their decomposition is decreased. This, in turn, triggers the role of QS in tissue relief and improved body performance (McGrath, 2007).

Considering the important role of sleep in neonates, it is possible to assume the extent to which deprivation of sleep can impose non-compensable problems for the growth of neonates in the future. Since neonates’ brain grows rapidly, those with neural damages in the form of Periventricular leukomalacia (PVL) had shorter sleep-wake intervals. The more intensive PVL is, the shorter the sleep-wake interval. Different interventions can improve neonatal sleep (both in term and preterm neonates). Studies are being conducted to provide such interventions in a better manner. Paying attention to neonates’ sleep in the NICU plays an important role in improving the quality of life of such sensitive neonates.

Carla Patton (2015) trained sleep-inducing methods to those mothers and nurses who were in contact with neonates. However, she developed sleep-inducing policies for NICUs in accordance with American Academy of Pediatrics (AAP) recommendations. She concluded that the vast majority of NICU nurses disagree with AAP recommendations and did not apply safe sleep measures on neonates. If mothers tried to practice the prone position on their neonates (lying with the face downward) the nurses would oppose this. Therefore, parents would try to discharge their child from the hospital more quickly due to this reaction of nurses. Some nurses would not practice the supine position and parents used to do the same mistake too.

Bullock et al. (2004) conducted study in baby friendly hospitals which revealed that 96% of nurses were informed of AAP recommendations and 45% of them believed that the prone position increased the risk of aspiration. A study by Hein and Pettit (2001) showed that 51% of nurses recommended side-lying to avoid aspiration. A study by Delzell et al. (2001) trained different sleep-inducing positions to nurses and mothers, including the prone position which is the best position for inducing sleep in preterm neonates. Despite the trainings, a small number of nurses practiced such positions. Protecting neonatal sleep in the NICU is an essential element of developmental care in term and preterm neonates (Calciolari & Montirosso, 2011). Newborn Individualized Developmental Care and Assessment Program (NIDCAP) proposed by Als et al. is the best and the most fit developmental care and assessment program for neonates. It help nurses to provide neonates with the best fit care solution based on the behavior of neonates in order to improve neonatal sleep (Als et al., 1994).

When preterm neonates (fetus outside uterus) are admitted to the NICU, they become deprived of the ideal sense of amniotic fluid of uterus as well as sleep. Therefore, they have no choice but to tolerate environmental stimuli and manipulation as well as medical and nursing cares. This means that preterm neonates should not be manipulated so much in order to prevent sleep deprivation. All nurses need to be aware of sleep stages, benefits of sleep and its disturbing and inducing factors. This study was conducted in the NICU in order to investigate NICU nurses about neonates’ sleep state.

## 2. Materials and Methods

This descriptive study was conducted in Ali-ibn-Abitaleb Hospital of Zahedan University of Medical Sciences in 2014. A total number of 30 nurses working in the NICU and neonate units participated in the study through the census method. Inclusion criterion was having more than one year of experience in neonatal units and the NICU. Two questionnaires were used in this study. The first one was for demographic information including gender, age, education and occupation and the second one involved measuring sleep. The latter was extracted from a questionnaire translated by Rahimi and was used after early implementation and obtaining internal validation and correlation. The validity of the questionnaire was confirmed by related specialists and supra-specialists after reviewing similar studies. Its reliability, however, was calculated through a primary study using Cronbach’s alpha coefficient formula and calculated 92%. This questionnaire had 10 questions scored as Likert scale points. Participants scored the questions by assigning a point from 1 to 4. The score scope ranged from 1 to 40.

Numbers between 30 to 40, 21 to 30, 19 to 20, 10 to 19 and below 10 stand for very high, high, moderate, weak and very weak awareness, respectively. The researcher was present at different shifts (Morning, Evening, night) of the study and asked nurses to fill the questionnaire out. Each questionnaire was completed within 15 minutes. There were different questions in the questionnaire about noise level in the NICU, different sleep cycles of neonates, specifications and importance of AS in neonates, factors contributing to sleep deprivation in neonates, methods for inducing sleep in term and preterm neonates, role of sleep in neural detoxification, use of Kangaroo mother care (KMC) for inducing sleep in neonates and reducing noise level.

When questionnaires were collected, the data tabulated and entered in Social Package for Social Sciences (SPSS) version 16.0 and was analyzed by expert. In analyzing, descriptive statistics methods were used.

## 3. Findings

The age of nurses ranged from 27 to 40 years old with a mean of 30-35 years old (76%). The experience of nurses in the NICU ranged from 5 to 20 years with 5 to 10 years as the most frequent experience (40%) (Table 1). Of the studied samples, 16% had a very good awareness (scored from 30 to 40), 24% had a good awareness (scored from 21 to 30), 28% had a moderate awareness (scored from 19 to 20) and 28% had a weak awareness (scored from 10-19).

**Table 1:** NICU nurses demographic data

Sex (female)	Number (30)	100%
B.S	26	73%
Diploma	4	13%
Age (25-30) years old	5	16%
Age (30-35) years old	23	76%
Age (35-40) years old	2	6.6%
Experience in the NICU (<5 years)	3	10%
Experience in the NICU (5-10 years)	12	40%
Experience in the NICU (10-15 years)	5	16%
Experience in the NICU (15-20 years)	10	33%

The vast majority of the cases gave a correct answer to questions about sleep-inducing factors in the NICU (72%), ideal noise level in the NICU (92%), decreased light and KMC (76%) and standard methods for sleep description (68%). However, 68%, 79.2%, 70.8% and 52% of cases gave an incorrect answer to questions about sleep cycles, AS specifications, sleep differentiation in fetus and the role of sleep in neural detoxification, respectively. In addition, 24%, 20%, 48% and 92% of cases gave a correct answer to questions about sleep cycles, AS specification in neonates, the role of sleep in saving energy and ideal noise level. See the Table 2 for NICUs nurses’ awareness of neonatal sleep in the NICUs.

**Table 2:** Questionnaire for NICU nurses’ awareness of neonatal sleep in the NICUs

What is the ideal noise in the NICU? A) Below 55 dB B) Below 45 dB C) Below 65 dB D) Below 75 dB

Which one is not a component of neonates’ state system? A) Sleep-wake cycles B) Wake level of neonates C) Experience of neonates D) Physiological changes

In which intrauterine age of human fetus are sleep stages differentiated? A) 26 weeks B) 27 weeks C) 28 weeks D) 30 weeks

Which one is included in neonatal sleep cycle? A) Two QS and AS phases B) Two REM and NREM phases C) One QS phase and one NREM phase D) There QS, REM and NREM phases

As a neonate grows older, the duration of …. sleep decreases while the duration of …. sleep increases A) QS, REM B) NREM C) QS, REM D) QS, AS

Which indicates specifications of REM in neonates? A) Closed eyes, relatively regular and abdominal breathing, relatively strong muscle tone B) Closed eyes, irregular and abdominal breathing, weak muscle tone C) Closed eyes, irregular and abdominal breathing, relatively strong muscle tone D) Closed eyes, relatively regular and abdominal aspiration, weak muscle tone

In which sleep doesn’t have a role? A) Brain development B) Energy saving C) Increased body temperature D) neural detoxification

…. Is the standard method for studying and describing the sleep A) Observation B) Observation with designed tools C) Polysomnography and observation D) Polysomnography

Which is not included among factors depriving neonates of sleep in the NICU? A) Too much light B) Noise C) Kangaroo care D) painful procedures

Which supports neonatal sleep in the NICU? A) Developmental care B) Kangaroo care C) Speaking in the NICU with a volume as low as speaking in the library D) All cases


## 4. Discussion

Any man has needs that should be met. Some of them are essential, crucial and physiologic. Sufficient sleep is a physiological need required for normal development (Dang-Vu et al., 2006). However, it is considered as an important neonatal behavior. Therefore, care nurses should pay special attention to it. During recent decades, the concept of developmental care has been introduced to improve the development trend of neonates, especially the development of central nervous system (CNS). Considering the importance of sleep in the neonatal period, Bertell *et al*. conducted a review study to investigate the benefits of sleep as well as neonatal sleep care methods in the NICU. The study emphasized the influence of developmental care on neonatal sleep (Blackburn, 1998). A fit response to the behaviors of neonates constitutes the fundamental of developmental care with neonates’ sleep as an important behavior. Developmental care interventions serve as the supplement of medical cares in the NICU. Protecting neonates’ sleep is among the benefits of developmental care intervention.

According to conducted researches by Dutta and Ramachandran, KMC is a recommended method for increasing duration of sleep in neonates (Ramachandran & Dutta, 2013). In this position, the neonate is lying downward on the mother’s/father’s abdomen and touches the mother’s/father’s skin. KMC rises sleep duration, especially QS sleep (Ludington-Hoe et al., 2008; Ludington-Hoe et al., 2004). It is generally practiced in preterm neonates when the fetus is 32 weeks old and extended to the time of discharging from the hospital. It results in the improved sleep-wake cycles in term and preterm neonates. This is another sign of the positive effect of KMC on brain development (Scher et al., 2009; Feldman et al., 2002). Skin to skin contact (SSC), and massage are interventions improving neonatal sleep with a positive effect on the primary development of brain (Im, Kim, & Cain, 2009; Guzzetta et al., 2011). According to Harrison et al., (2000) gentle human touch (GHT) improves the duration of QS sleep. Massaging neonates when they are wake improves neonatal sleep (Ramachandran & Dutta, 2013). The studied cases used to employ KMC only to induce effective sleep so that 87% of cases believed that KMC is a way for inducing sleep in neonates and used to practice KMC in preterm neonates.

Sleep position is another factor affecting sleep/wake state of neonates, especially in preterm ones (Allen, 2012). Side lying rises QS sleep time (Liaw et al., 2012). Fetus position *i.e*. bent organs and body improves neonatal sleep.

This study agrees with a study by Patton et al. (2015). They reported that nurses have a little information about neonatal sleep in that NICU so that two studies out of 13 reported insufficient information about proper sleep positions and seven researches emphasized the need of nurses for sleep positions, sleep policies and SISD prevention trainings (Patton et al., 2015; Coughlin, Gibbins & Hoath, 2009). Delzell et al. (2001) reported in their study that training programs should concentrate on pre-sleep positions. They concluded that nurses need to know sleep positions to be applied on neonates in order to provide them with sufficient sleep. According to the study, nurses’ awareness of sleep-inducing factors was weak. The results in this study are in line with our study. Moon & Omron (2002) conducted a study in which nurses put barriers around neonates in supine position in order to provide sufficient sleep. Following necessary trainings, the nurses would be able to train parents to provide sufficient sleep for their neonates. This finding is in line with our study. In addition, our study reported an insufficient awareness of nurses of preterm neonates’ sleep as well as standard methods for inducing sleep. Light and noise are disturbing factors of neonatal sleep in NICU. Therefore, it can be argued that improving the environmental condition of NICU in terms of light and noise will improve neonatal sleep (Colombo & Bon, 2011). In that study, 69% of cases believed that reduced noise is an important factor of sleep-inducing. This research agrees with our study.

Neonatal care, especially preterm neonates, in low light improves sleep-wake patterns. A study revealed that reduced environmental light improves neonatal sleep through covering incubator (Bertelle et al., 2005; Hellstrom-Westas et al., 2001). Protecting neonates’ eyes from direct light can help the improvement of sleep cycles (Graven 2011). During phototherapy, for example, neonates’ eyes should be covered. During night silence period, the light should be decreased as much as the noise (Bertelle et al., 2005). As it was mentioned before, the noise in the NICU should be reduced in order to improve neonatal sleep. Behavioral methods can help achieve this goal which include training NICU employees to speak in a laboratory acceptable voice i.e. below 45 dB, to speak far from neonates and to keep neonates’ environment quiet as much as possible so that the neonates can hear their mothers (Laudert et al., 2007). In our study, 96% of cases believed that the noise in the NICU should be at the level of speaking in library. Rao et al. (2007) studied 182 NICU nurses and concluded that they should be trained about safe sleep measures based on AAP guidelines. They gave necessary trainings to the nurses and reassessed their awareness in 2007 and reported the rise of their awareness of neonatal sleep. Our study, however, reported a less awareness of nurses of neonatal sleep and concluded that they should be provided with necessary trainings about inducing sleep in neonates.

Grazel et al. (2010) conducted a study and suggested that if nurses are trained about safe sleep, the risk of sudden infant death syndrome (SIDS) in neonates decrease. Patton et al. (2015) reported an unbalanced awareness of nurses of safe sleep. Some had sufficient awareness but did not use it. Some had less awareness of sleep positions and had trained nothing to the parents of preterm neonates. Peeke et al. (1999) reported that 97% of nurses were informed of AAP guidelines but only 67% of them agreed with them whereas 29% of them used the supine position. They observed that the vast majority of nurses use the side-lying position. They did not use the supine position neither in term nor preterm neonates. In our study, however, nurses only used KMC to induce effective sleep. In their study, Aris et al. (2015) gave necessary trainings to nurses about safe sleep. The nurses, in turn, trained preterm neonates’ parents. After 2 years, they reassessed the awareness of nurses. This time, they observed that the nurses use the supine position for neonates with respiratory problems. The researchers of 4 other studies who had trained the parents about safe sleep position observed that the vast majority of participants apply this position and believe that it is an effective position. They concluded that all nurses should be continuously trained about safe sleep in order to apply this theory in clinical positions and train, in turn, the parents of neonates. If nurses and mothers use proper position to induce sleep in neonates, neural and brain development improves (Aris et al., 2006). The results of this research showed that the cases have negligible information about the important role of sleep in neural detoxification as well as neural and brain development.

## 5. Conclusion

Every man has needs, some of which are essential and should be met. Sufficient sleep is one of the most vital neonatal needs. Sleep is considered an important neonatal behavior and care nurses should pay sufficient attention to it.

Relationship between neonates’ sleep and long-term consequences and the quality of life in future highlights the important role of nurses in protecting and improving neonatal sleep in the NICU.

NICU neonates, especially preterm ones, are in rapid brain growth stage where there is a strong relationship between sleep and brain development. Therefore, special attentions should be paid to neonatal sleep in the NICU. Preterm neonates are separated from their mothers and exposed to environmental stimuli in the NICU like light, noise and different stresses. On the other hand, NICU nurses spend most of their time with preterm neonates. Therefore, they should pay a special attention to behavioral responses of neonates, especially sleep. However, it is necessary to pay more attention to nurses training about neonatal sleep. The trainings should follow American Nurses Association (ANA) guidelines and should be practiced continuously. Nurses need to use all of their knowledge beside neonates. The use of questionnaire was a limitation of this study. It would be better to ask the questions in the form of interview.

**Figure 1:**
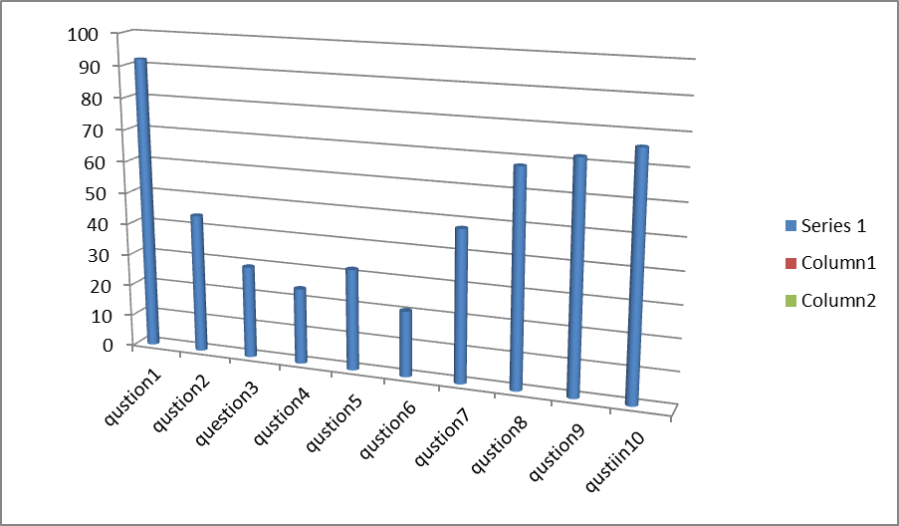
3= correct answer
